# Differentially Methylated Super-Enhancers Regulate Target Gene Expression in Human Cancer

**DOI:** 10.1038/s41598-019-51018-x

**Published:** 2019-10-21

**Authors:** Emily L. Flam, Ludmila Danilova, Dylan Z. Kelley, Elena Stavrovskaya, Theresa Guo, Michael Considine, Jiang Qian, Joseph A. Califano, Alexander Favorov, Elana J. Fertig, Daria A. Gaykalova

**Affiliations:** 1 Department of Otolaryngology—Head and Neck Surgery, Johns Hopkins Medical Institutions, Baltimore, Maryland USA; 2 Division of Oncology Biostatistics and Bioinformatics, Department of Oncology, Johns Hopkins Medical Institutions, Baltimore, Maryland USA; 3 Laboratory of Systems Biology and Computational Genetics, Vavilov Institute of General Genetics, Russian Academy of Sciences, Moscow, Russia; 4 Department of Bioengineering and Bioinformatics, Moscow State University, Moscow, 119992 Russia; 5 Institute for Information Transmission Problems, RAS, Moscow, 127994 Russia; 6 Department of Ophthalmology, Johns Hopkins Medical Institutions, Baltimore, Maryland USA; 7 Department of Surgery, Head and Neck Cancer Center, University of California, San Diego, California USA

**Keywords:** Gene expression analysis, Data integration, Histone post-translational modifications, Head and neck cancer

## Abstract

Current literature suggests that epigenetically regulated super-enhancers (SEs) are drivers of aberrant gene expression in cancers. Many tumor types are still missing chromatin data to define cancer-specific SEs and their role in carcinogenesis. In this work, we develop a simple pipeline, which can utilize chromatin data from etiologically similar tumors to discover tissue-specific SEs and their target genes using gene expression and DNA methylation data. As an example, we applied our pipeline to human papillomavirus-related oropharyngeal squamous cell carcinoma (HPV + OPSCC). This tumor type is characterized by abundant gene expression changes, which cannot be explained by genetic alterations alone. Chromatin data are still limited for this disease, so we used 3627 SE elements from public domain data for closely related tissues, including normal and tumor lung, and cervical cancer cell lines. We integrated the available DNA methylation and gene expression data for HPV + OPSCC samples to filter the candidate SEs to identify functional SEs and their affected targets, which are essential for cancer development. Overall, we found 159 differentially methylated SEs, including 87 SEs that actively regulate expression of 150 nearby genes (211 SE-gene pairs) in HPV + OPSCC. Of these, 132 SE-gene pairs were validated in a related TCGA cohort. Pathway analysis revealed that the SE-regulated genes were associated with pathways known to regulate nasopharyngeal, breast, melanoma, and bladder carcinogenesis and are regulated by the epigenetic landscape in those cancers. Thus, we propose that gene expression in HPV + OPSCC may be controlled by epigenetic alterations in SE elements, which are common between related tissues. Our pipeline can utilize a diversity of data inputs and can be further adapted to SE analysis of diseased and non-diseased tissues from different organisms.

## Introduction

Super-enhancers (SEs) are tissue- and disease-specific regulatory genomic elements related to chromatin that drive cell-specific gene expression changes in development, differentiation, and disease progression, including cancer^1,2^. SEs are enriched for binding of many transcription factors, as well as Mediator, RNA polymerase II, and BRD4 proteins, which they bring to the promoter regions of *in cis* target genes through the formation of chromatin loops^2–7^. SEs are marked by specific histone modifications, such as H3K27ac and H3K4me1^3,8^, suggesting an essential role of the chromatin landscape in SE-mediated gene expression regulation. SEs can cover up to 300 kb regions^9^ and influence the expression of genes with transcription start sites (TSS) up to 1.5 Mbp away^3,10^. Genes regulated by SEs are more expressed than those regulated by typical enhancers and are often associated with tissue-specific or disease-specific cell-identity^2,3,11^. Moreover, the chromatin landscape predetermines disease-specific genetic alterations in cancer^12^. SEs can appear *de novo* during carcinogenesis in proximity to their cancer-related gene targets, causing changes in the relative gene expression of multiple genes simultaneously^13,14^.

Differential methylation has been used as a hallmark for SE detection^8,15–18^. Therefore, it is critical to study the interplay between DNA methylation of SEs and their effects on the regulation of target gene expression. SE methylation can be altered during the carcinogenesis process, independent of global disease-specific methylation changes in cancer cells^8,16^. We propose that hypomethylation of SE regions causes increased expression of nearby oncogenic target genes and that hypermethylation of these SEs causes decreased expression of targets, especially tumor suppressors^8,15,19^.

SEs can be detected through chromatin-focused studies, such as chromatin immunoprecipitation with high throughput sequencing (ChIP-Seq)^3^ or assay for transposase-accessible chromatin with high throughput sequencing (ATAC-Seq)^13^. While SEs are currently recognized as one of the main drivers of carcinogenesis, many tumor types are still missing information about SE locations. This lack of information can be explained by the challenges of chromatin analysis procedures on clinical biopsy tissues^20^. Nevertheless, annotation and characterization of the SEs based on H3K27Ac chromatin data have been presented in numerous cancer cell lines^2^. We hypothesized that etiologically similar tumors might share a portion of SE regions to regulate similar genes due to the genetic and epigenetic similarities noticed between certain tumor types^13^. Therefore, it is possible to utilize chromatin-related data available for etiologically-relevant tissues for the discovery of SEs in other tumor types that still lack chromatin data. This type of data is widely available for diverse tumor types through projects like The Cancer Genome Atlas (TCGA) and can be used to navigate through SE candidates from etiologically relevant samples.

**Figure 1:**
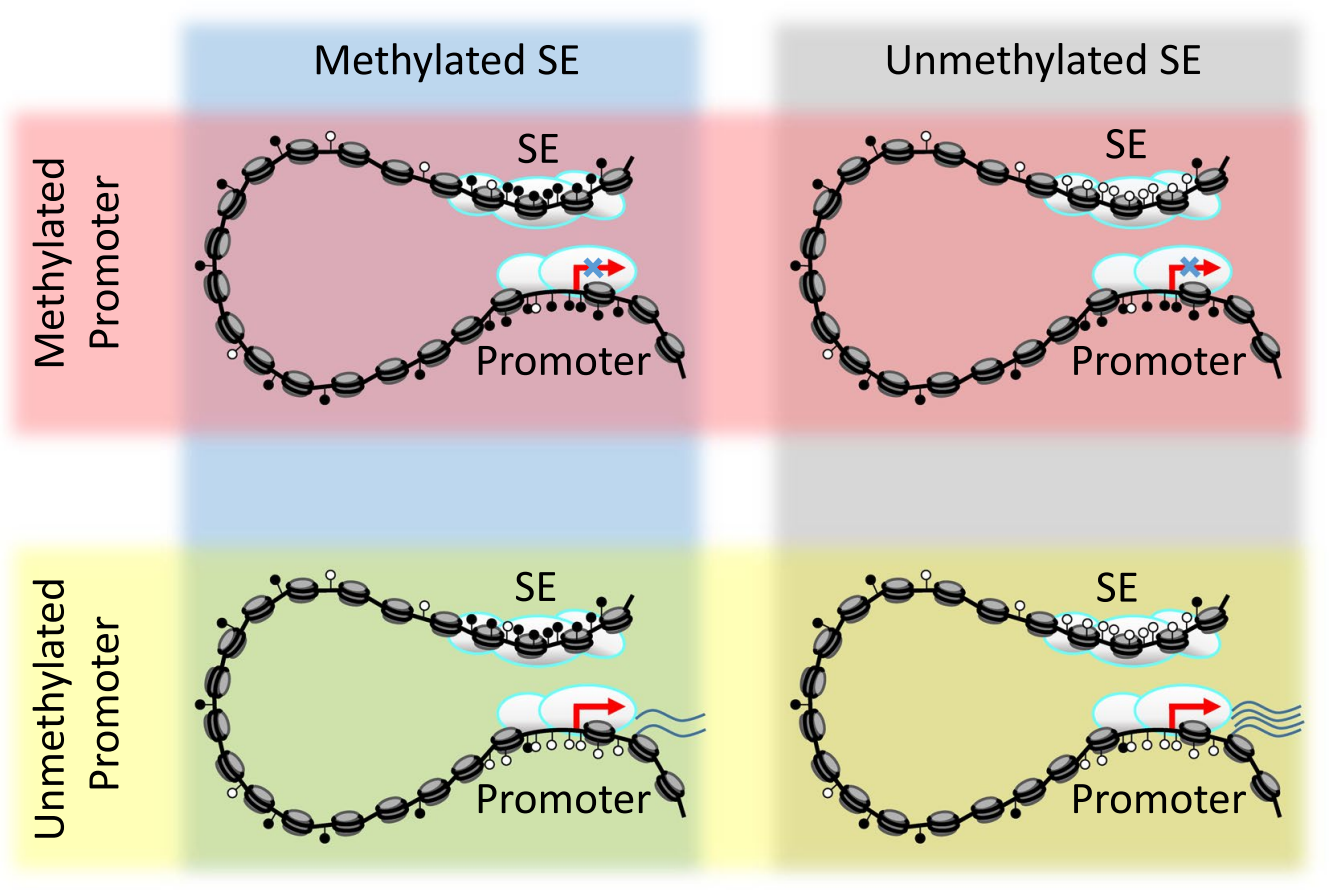
Scheme of how DNA methylation of either promoter or SE can affect target gene expression. Methylation at the promoter region prevents the expression of a gene, regardless of SE methylation. Genes can be minimally expressed with an unmethylated promoter, even with a methylated SE region, but maximal gene expression is reached with both an unmethylated promoter and an unmethylated SE.

In this study, we introduce a pipeline to detect SE regions by using gene expression and DNA methylation for a particular set of samples. This pipeline helps to define the role of SE methylation in target gene expression in human carcinomas. We built our pipeline under the assumption that maximum gene expression occurs under the condition that both the gene promoter region and nearby SE region are hypomethylated, while hypermethylation signal from either the promoter or the SE region could diminish target gene expression (Fig. 1 and refs^8,15,19^).

As an example of the power of our pipeline, we studied high-risk human papillomavirus-related oropharyngeal squamous cell carcinoma (HPV + OPSCC), which has limited available chromatin data. We chose this model because the development of HPV + OPSCC cannot be fully explained by its mutational landscape alone^21–25^. Most mutations in OPSCC are found in tumor suppressors^21–23,25,26^ and are coupled with pervasive genome-wide alterations to DNA methylation, but these alterations are still insufficient to explain the widespread gene expression changes observed in HPV + OPSCC^21,27,28^. Mutations in chromatin-related genes have been implicated in head and neck carcinogenesis, including K27 and K36 of the histone 3 tail^28–31^. Given the extensive epigenetic changes in HPV + OPSCC, we hypothesized that methylation of SEs is a critical driver of transcriptional changes in carcinogenesis. We utilized our pipeline to identify actionable SE elements using ChIP-Seq data published for lung and HPV + cervical cell lines^2^, which are all closely related to HPV + OPSCC^13,21^. We hypothesized that SE regions are conserved between these tissues and HPV + OPSCC, resulting in transcriptional activation of target genes in HPV + OPSCC.

Using our pipeline, the candidate SE regions from lung and HPV + cervical samples were filtered through the integrated analysis of gene expression and DNA methylation patterns in our JHU cohort of HPV + OPSCC^32,33^. DNA methylation of gene promoters may also impact gene expression. Therefore, we have also evaluated the methylation status of promoter regions on SE-regulated genes. We identified 211 gene-SE pairs in HPV + OPSCC, of which we have validated n = 132 in the HPV + OPSCC cohort from TCGA project^21^. These critical SE regions suggest epigenetic regulation as a mechanism for HPV + OPSCC carcinogenesis.

We believe our pipeline can be adopted for SE filtering and analysis of other cancer types, non-cancer diseases, and other tissue types for any organism with available data. The pipeline can use SEs for the same tissues or for etiologically-similar samples and can incorporate a range of relevant data input from various platforms.

## Results

### Pipeline to identify differentially methylated SE that regulate target gene expression

**Figure 2:**
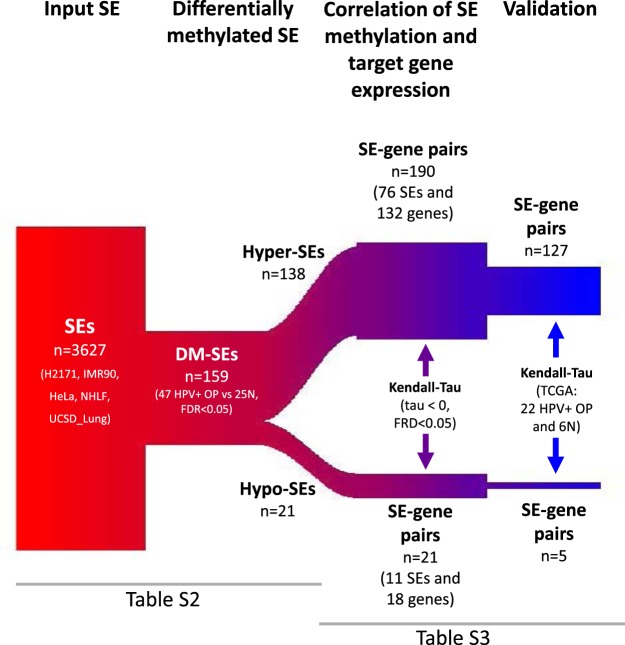
Experimental scheme. Pipeline for analysis of SEs, including SE input and initial filtering steps, detection of differential methylation of SEs, correlation of target gene expression with SE methylation, and validation of results.

We developed a pipeline to study the role of SE methylation on target gene expression. The pipeline takes a list of SEs as an input, which can vary depending on the study question, cancer type of interest, data availability, etc. The main steps of the pipeline for the analysis of HPV + OPSCC’s SEs are shown in Fig. 2 and include differential methylation analysis of SE and correlation analysis of SE methylation with target gene expression. While we used HPV + OPSCC as an example throughout the manuscript, the pipeline can be applied to any cohort that has DNA methylation and gene expression data available for the same samples. As an output, the pipeline creates SE-gene pairs where methylation of the SE region significantly correlates with target gene expression. This set of pairs can be validated in different cohorts and be supplemented with pathway analysis, functional analysis, correlation analysis, experimental analysis, and so on. The R code for the pipeline is available through this link: https://bitbucket.org/favorov/cervical-lung-se-and-hnscc/downloads/ and in the supplementary information.

### Differentially methylated SEs regulate target gene expression in human papillomavirus-related oropharyngeal squamous cell carcinoma

To demonstrate a possible use for our pipeline, we applied it to find differentially methylated SEs that regulate gene expression in HPV + OPSCC.

#### Input SEs

As an input list of candidate SEs, we obtained a pooled list of SEs for lung cancer (H2171), non-cancer lung (NHLF and IMR90), and HPV + cervical cancer (HeLa) cell lines and healthy lung tissue (UCSD_Lung) available from a previous study^2^ (Table S1) for a total of 3627 candidate SEs (Table S2, Fig. 2). To evaluate whether these candidate SEs were conserved across tissues, we calculated the Jaccard index of 3627 SEs from the five cell lines described above and 54656 SEs from the remaining 81 cell lines available from the same study^2^ using the Genometricorr package^34^. We obtained a Jaccard index of 0.17, which means the two sets of SEs had a small intersection, which suggests high conservation between our chosen study input SEs relative to all other tissue types and their tissue- and disease-specificity.

#### Differentially methylated SE

**Figure 3:**
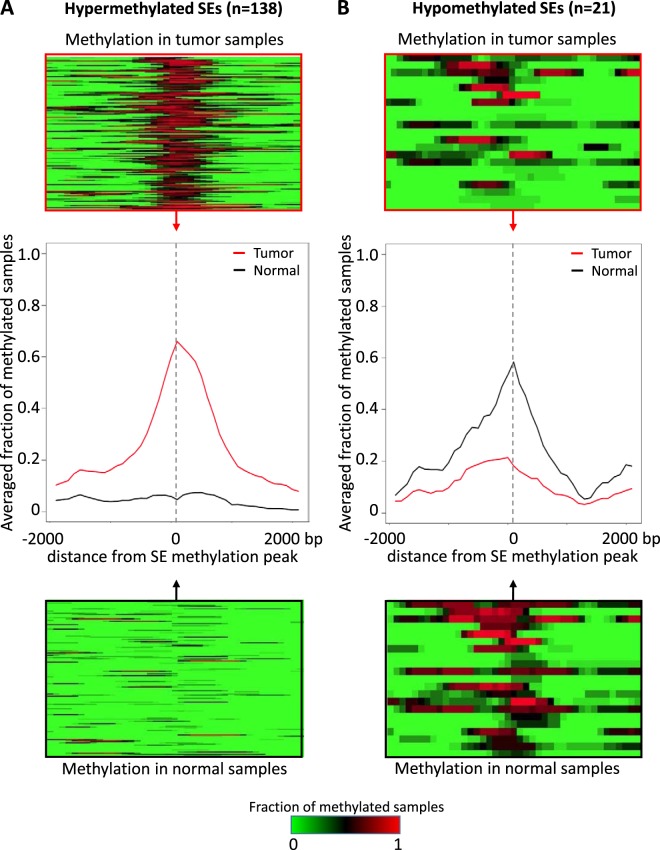
Methylation landscape of DM-SEs. Individual and averaged JHU cohort methylation of the tumor (top, red) and normal (bottom, black) samples across the SE region for (**A**) hypermethylated and (**B**) hypomethylated SEs.

**Figure 4:**
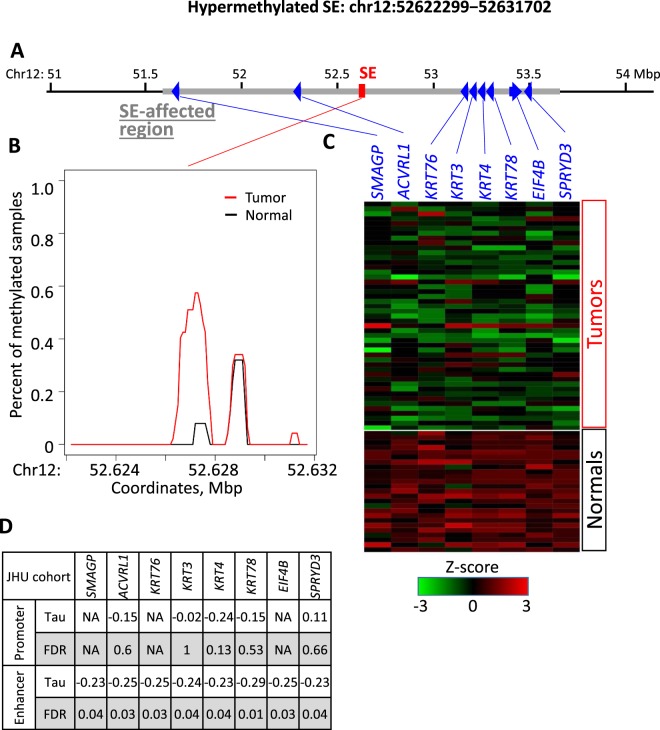
Methylation and genetic landscape of the representative hypermethylated SE: chr12:52622299−52631702 in JHU cohort. (**A**) Genomic landscape of the SE region and potential target genes within one Mbp of the SE. (**B**) Relative average methylation coverage across the SE region (red – tumor, black – normal). (**C**) Log-transformed RNA expression of the potential target genes (z-score). (**D**) Kendall-tau values and corresponding FDR for correlation of promoter methylation with gene expression, as well as SE region methylation with gene expression of target genes.

**Figure 5:**
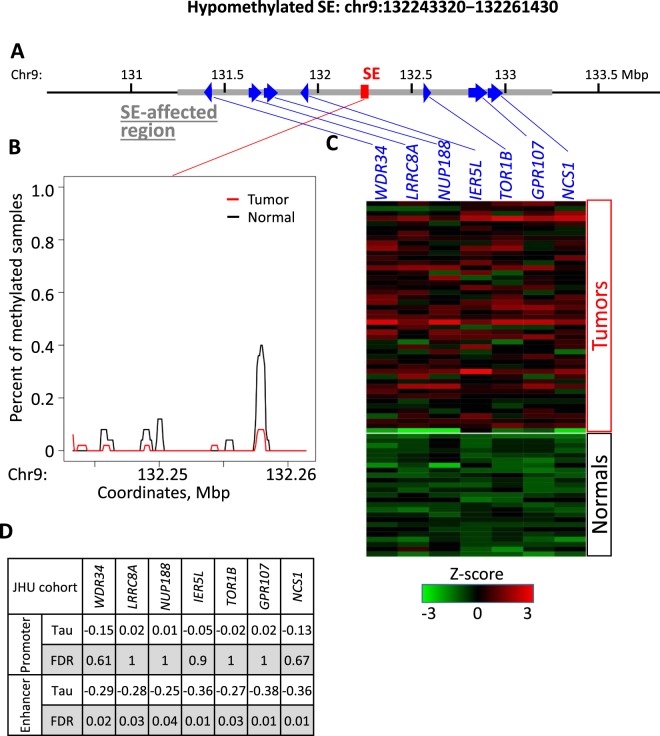
Methylation and genetic landscape of the representative hypomethylated SE: chr9:132243320−132261430 in JHU cohort. (**A**) Genomic landscape of the SE region and potential target genes within one Mbp of the SE. (**B**) Relative average methylation coverage across the SE region. (**C**) Log-transformed RNA expression of the potential target genes. (**D**) Kendall-tau values and corresponding FDR for correlation of promoter methylation with gene expression, as well as SE region methylation with gene expression of target genes.

We analyzed whole-genome DNA methylation in a previously published cohort of n = 47 HPV + OPSCC samples from tumor patients and n = 25 normal mucosal samples from uvulopalatopharyngoplasty surgery patients (UPPP)^20,35^. In this cohort, 0.07% of the genome was differentially methylated in tumor samples relative to normal controls. To validate the correlation between SE location and differential methylation in HPV + OPSCC, we compared the distribution of differentially methylated 100 bp regions relative to candidate SE sites. Differentially methylated regions were significantly overrepresented in the 3627 SEs (GenometriCorr analysis^34^: observed/expected ratio = 3, p-value < 10^−16^) compared to background DNA. Using the Wilcoxon rank sum test, we found that 159 (4%) of 3627 SE candidates were differentially methylated (DM-SEs) between tumor and normal samples in our cohort (FDR < 0.05, Table S2, Figs 2 and 3). These SEs included 138 (87%) SEs that were hypermethylated (Figs 3A, 4A-B and S1A) and 21 (13%) SEs that were hypomethylated (Figs 3B, 5A,B and S2A).

#### Correlation of SE methylation and target gene expression

To test whether differential methylation of SEs regulates the expression of at least one *in cis* target gene, we gathered all genes with TSS within 1 Mbp of each DM-SE region. Overall, we detected 2,675 genes to be potentially affected by 159 DM-SEs, suggesting that each DM-SE regulates an average of 28 genes (range: 1–91 genes, Table S2). Target genes were located an average of 471,800 bp (range: 20–1,102,000 bp) from the SE region (Fig. S3). For each of the DM-SE-gene pairs, we correlated the methylation of SEs with the expression of target genes using Kendall’s tau test. A statistically significant negative correlation (FDR < 0.05) between gene expression and SE methylation was confirmed for 211 SE-gene pairs (Tables S3). Of these pairs, 190 SE-gene pairs (93%) were linked to hypermethylated SEs and 21 pairs to hypomethylated SEs. The overrepresentation of hypermethylated SE-gene pairs is consistent with OPSCC being a tumor suppressor-dysregulated disease^36^.

Hypermethylation of SE regions has been linked to silencing of target genes^15^. Of the 138 DM-SEs, 76 (55%) had at least one target gene with decreased expression in cancer (Figs 2 and S4), totaling 190 SE-gene pairs and 132 individual genes (Table S3). An illustration of one representative hypermethylated SE and its target genes is provided in Fig. 4. For this SE, we found eight potential target genes that were under-expressed in tumors relative to normal samples (Figs 4C and S1B). The representative hypermethylated SE from Fig. 4 is linked to lower expression of *SMAGP* (Small Cell Adhesion Glycoprotein, Fig. S5A-B), which plays a role in epithelial cell-cell contact^37^.

Differential hypomethylation of SEs is linked to activation of oncogene expression^8,15,38^. We documented an increased expression of 18 genes linked to 11 (52%) out of 21 hypomethylated SE regions, which formed 21 SE-gene pairs (Fig. 2 and Table S3). An illustration of one representative hypomethylated SEs and its target genes is provided in Fig. 5. For this SE, we found seven potential target genes with overexpression in tumors relative to normal samples (Figs 5C and S2B). Hypomethylation of this SE is strongly linked to overexpression of *GPR107* (G Protein-Coupled Receptor 107, Fig. S5C,D), which is commonly overexpressed in breast cancer patients with worth prognosis^39^.

For the genes that had a significant correlation between expression and SE methylation, we also quantified the correlation of its expression with promoter methylation. Most promoters had little to no methylation, and only a small portion of SE-gene pairs showed strong correlation for both promoter and super-enhancer methylation (Figs 4D and 5D). These data emphasize the significant role of super-enhancer methylation on target gene expression.

#### Validation

To confirm the regulation of gene expression by methylation of the SE regions, we utilized TCGA data from 22 HPV + OPSCC and six normal samples with both DNA methylation and gene expression data^21^ to match our JHU cohort. Out of the 211 SE-gene pairs, 132 (63%) had a significant association between SE methylation and gene expression in the TCGA cohort (Table S3 and Figs S1 and S2). Of these 132 validated SE-gene pairs, five were regulated by hypomethylated SEs, and 127 were regulated by hypermethylated SEs (Table S3).

As an additional step of validation, we performed gene set analysis on 132 target genes of hypermethylated SEs. The target genes, whose expression was downregulated in cancer, belong to gene sets such as BREAST CANCER NORMAL LIKE UP, NASOPHARYNGEAL CARCINOMA, DIFFERENTIATING T LYMPHOCYTE, BOUND BY FOXP3, as well as epigenetic related BRAIN HCP WITH H3K4ME3 AND H3K27ME3, ES ICP WITH H3K4ME3, HDAC TARGETS SILENCED BY METHYLATION DN, and many more (Table S4, top). Eighteen upregulated genes linked to hypomethylated SEs were related to BLADDER CANCER WITH LOH IN CHR9Q AND UVEAL MELANOMA UP (Table S4, bottom). This analysis demonstrates that the detected gene targets belong to gene sets that play a significant role in carcinogenesis.

## Discussion

In this study, we developed a new bioinformatics pipeline to define and evaluate differentially methylated SEs that regulate target gene expression. Depending on the scientific question, data availability, or disease, the pipeline can be applied to any list of SEs or other defined DNA region, including from other organisms and experimental models. The code for the pipeline is publicly available and can be adapted to use on any cohort of samples that have parallel DNA methylation and gene expression data available. In this paper, we demonstrated how our algorithm integrates methylation and expression data with a list of potential SE sites for the discovery of functional, tissue-specific SEs in OPSCC. The algorithm can use a list of SEs identified from the same study samples or from etiologically-similar samples, which is especially valuable for samples that have limited chromatin data. Indeed, our results suggest that biological signatures in one cancer type can be detected and validated using SE data from related cancer types.

The input SE list goes through the stringent sorting process. DNA methylation data from study samples is the primary filter, which helps to remove all non-phenotype-related SE candidates. This assumption is built on recent works demonstrating that actionable SEs in a particular tissue are expected to be differentially methylated^8,16^. Previous studies developed algorithms to predict functional, tissue-specific SEs by applying machine learning algorithms to integrated DNA methylation and SE data^17^. Therefore, the incorporation of such methylation status of SEs regions increases the accuracy and biological relevance of our SE predictions. Our pipeline for this step of the algorithm is based on the well-known MACS peak-calling procedure. Interestingly, in our examples, the full differential signal is provided by local differentially methylated regions (Figs 4, 5, S1, and S2). It is concordant with the work of others^40^ that SEs and their parts (individual typical enhancers) both carry the regulatory signal. Still, we do not explicitly relate the unsupervised uniformed probe regions we use with specific SEs. Furthermore, in the future, we are going to compare with modern algorithms, such as MBDDiff^41^.

The application of our pipeline allowed us to define tissue-specific SE candidates in HPV + OPSCC, their methylation status, and its correlation with target gene expression. This tissue type has minimal chromatin data available, and SEs for this disease are not yet described. Although many SEs are disease- and tissue-specific^1,2,5,38^, similar genetic profiles between head and neck, cervical, and lung cancers^21^ suggest conservation of SE regions between these diseases and tissues. We observed many examples of analogous SEs from two or more different cell lines, suggesting the presence of pan-SEs that are common across tissues types and disease status. Our findings were consistent with recent data, which suggest that a subset of multiple myeloma-specific SE candidates was differentially methylated in six head and neck primary samples (three HPV + and three HPV-)^42^. The current study presented a high confidence list of candidate head and neck SEs that were detected in cervical and lung tissue and were differentially methylated in HPV + OPSCC samples. These SE had statistically significant links to gene expression of their *in cis* targets, which participate in cancer-relevant pathways. Such work provides the groundwork for the future discovery of novel, HPV + OPSCC-specific SEs.

We identified 21 differentially hypomethylated SEs and 138 differentially hypermethylated SEs that were linked to the expression of *in cis* target genes in HPV + OPSCC. The prevalence of hypermethylated SEs suggests that hypermethylation of SEs leads to downregulation of normal cellular homeostasis. For example, the observed down-regulated genes are important in breast cancer (BREAST CANCER NORMAL LIKE UP; BREAST CANCER LUMINAL B DN). The significance of those SE-gene links was validated using one of the largest HPV + OPSCC cohorts with in-parallel gene expression and DNA methylation data available for the same samples from TCGA^21,32,33^.

We note several limitations of our study. First, the clinical characteristics between tumor and non-tumor groups are not matched in the JHU patient cohort^32,33^ due to the demographics of UPPP and OPSCC populations^43–46^, with differences in age and smoking status. Nonetheless, a similar UPPP population has helped to reveal strong cancer-specific signatures of OPSCC in previous studies^43–46^. Moreover, the employment of TCGA’s control population with matched clinical characteristics validated our original discovery of tissue-specific SEs in HPV + OPSCC. Utilization of DNA methylation array data (Illumina Infinium HumanMethylation450 BeadChip) in TCGA restricted our validation of methylation-expression correlations for both SEs and promoters in this cohort due to the limited number of DNA methylation probes. The availability of probes weakens the correlation, as we are only able to link expression to methylation of one or a few coordinates in the SE region, as opposed to the entire region. The comprehensive discovery of SE candidates is possible only with MBD-Seq whole-genome data, as is employed in our JHU HPV + OPSCC cohort^32,33^, which is the only HPV + OPSCC cohort with MBD-Seq data available in parallel with RNA-Seq data for the same samples. The observed differences between the JHU and TCGA cohorts are expected, as normal TCGA samples are not from healthy patients, but are adjacent to the tumor sites of oral cavity tumor patients. These adjacent tissues are known to carry genetic and epigenetic alterations characteristic of OPSCC, skewing analyses^47^. Moreover, we could not define a correlation with survival because only three patients in the discovery cohort recurred in the last five years. Lastly, none of the SE candidates were functionally evaluated within the scope of this study, but they can be assessed in future work, which will also define the OPSCC-specific transcription factors associated with the SE regions.

In conclusion, we developed a new bioinformatics pipeline to define and evaluate SE activity in individual tissue types, which can be further adapted for a wide range of tissues to facilitate analysis of epigenetic mediators. Our pipeline and the SE candidates presented here provide an important next step in developing novel epigenetic therapies and biomarkers for detection of a variety of diseases.

## Materials and Methods

### JHU study cohort

The employed cohort of study samples is composed of 47 primary HPV + OPSCC tissue specimens and 25 control normal mucosal samples from uvulopalatopharyngoplasty (UPPP) surgeries of non-cancer-affected patients^32,33^. The clinical differences between tumor and control populations in this cohort were previously identified and acknowledged^32,33^. All tissue samples were obtained from the Johns Hopkins Tissue Core, as a part of the Head and Neck Cancer Specialized Program of Research Excellence (HNC-SPORE). These samples were acquired under the Internal Review Board-approved research protocol #NA_00036235. Informed consent was obtained from all patients recruited under this protocol prior to participation in the study. All methods for processing the high-throughput data for these samples were performed in accordance with the relevant guidelines and regulations.

### TCGA study cohort

The Cancer Genome Atlas (TCGA, http://cancergenome.nih.gov/cancersselected/headandneck) recently finished high throughput analyses of head and neck squamous cell carcinoma samples (HNSCC)^21^. The study analyzed data on 279 HNSCC tumors, including 35 HPV + tumors and 50 total adjacent non-cancer control tissues from the same HNSCC patients. Of the 35 HPV + HNSCC tumors, 22 samples were from the oropharynx, similar to JHU cohort, which were selected for cross-study validation to avoid introducing any cohort-specific biases in the analysis. Of 50 controls, we analyzed six samples confirmed as squamous epithelium tissues, while the other 44 TCGA samples designated as controls belong to muscle, salivary gland, and other tissues^21^. After the employed JHU cohort, TCGA is the largest HPV + OPSCC cohort with in-parallel analysis of DNA methylation and gene expression. All methods for processing the high-throughput data for these samples were performed in accordance with the relevant guidelines and regulations.

### Candidate SE regions

SEs were previously reported for NHLF (human lung fibroblast), IMR90 (human fetal lung), H2171 (small cell lung carcinoma), HeLa (HPV + cervical adenocarcinoma) cell lines, and UCSD_Lung (healthy lung tissue)^2^ (Table S1). Due to their genetic similarity to HPV + OPSCC^21^, this pooled list of SEs (n = 3627) was used as an input in our pipeline to look for SEs that are specific for HPV + OPSCC (Fig. 2, Table S2).

### Gene expression data and processing

RNA-Seq data was obtained for JHU^32,33^ and TCGA^21^ cohorts. JHU stranded RNA-Seq libraries from ribosomal RNA depleted total RNA were prepared using the Illumina TruSeq stranded total RNA Seq Gold kit and sequenced on the HiSeq 2500 (JHU) or HiSeq 2000 (TCGA) platform sequencer (Illumina) and the TruSeq Cluster Kit. RNA sequencing data from both cohorts were normalized based on the version 2 protocols developed by TCGA^21^. Gene expression values were quantified from RNA sequencing data using RSEM version 1.2.9 and upper quartile normalization according to the TCGA RSEM v2 normalization pipeline^21,32^.

### DNA methylation data and processing

#### MBD-Seq DNA methylation analysis for JHU samples

Genome-wide DNA methylation analysis was carried out using MBD-Seq (Methyl-CpG binding domain protein sequencing), as previously described^48,49^ using the NEBNext DNA Library Prep Set for the SOLiD sequencer. Methylated regions were identified as positional peaks of the population of aligned sequencing reads in the MBD-enriched data compared with the total input fraction using the MACS v1.4 software^50,51^. MACS builds an HMM model to identify peaks and indirectly takes the CpG density into account. This algorithm identifies peaks after accounting for both global and local biases using the enriched-to-input fraction. MACS p-value cut-off (p < 10^–6^) was used to identify reliable methylation peaks. Using the distribution of MACS-called DNA methylation regions, we performed a whole-genome identification of differentially methylated DNA regions between 47 HPV + OPSCC and 25 UPPP samples.

#### Illumina infinium humanmethylation450 array analysis of TCGA samples

Methylation array data were collected from the TCGA database. This platform includes probes for more than 480,000 CpG sites, spanning 99% of RefSeq. In total, 96% of CpG islands and 92% of CpG shores are represented by at least one probe. Beta values (percent methylation) were estimated from unmethylated (U) and methylated (M) measurements on a probe level basis: β = M/(M + U)^43,45^.

### Differential methylation analysis preparation

The methylation calculation was done by a function for intersection length calculations for a set of intervals (SEs, promoters) in multiple samples, which was provided by the “differential.coverage” R package^52^. Annotation functions, e.g., the enumeration of all genes with transcription start site around a SE, was also provided by this package^52^. The package was also used to prepare 100 bp probe intervals inside SE regions and to calculate their methylation for visualization (Figs 4 and 5**)**.

### Whole genome differential methylation by 100 bp regions

To identify differentially methylated genome regions using MACS-processed MBD-Seq data for 47 OPSCC and 25 UPPP (normal) samples, we separately tested 30,975,368 nonoverlapping regions of 100 bp each, which together completely cover the human genome. The methylation status of each 100 bp segment for each sample in the discovery cohort was determined as the presence of any intersection of the segment with regions of DNA methylation, as identified by MACS peak calling^50^ for that sample. The differentially methylated probes between diseased and normal phenotypes were identified by exact Fisher test for association of the probe methylation status with the sample status, followed by FDR correction.

### GenometriCorr analysis

We used overlap statistics provided by the GenometriCorr package^34^ to compare the genome-wide distribution of differentially methylated 100 bp regions relative to candidate SE sites and to test the conservation of the input list of 3627 SEs.

### SE differential methylation detection

Methylation of a SE region in each sample was identified as the length of the intersection of the SE region with methylation peaks provided by the MACS software for each sample^50,51^. MACS identifies peaks after accounting for both global and local biases using the enriched-to-input fraction. Using MACS-processed MBD-Seq data, we calculate the net length of methylated regions that overlap with each of the enhancers in each of the samples. For each SE, we calculated the Wilcoxon p-values for the difference of the methylation in the SE region between sample types of cases (n = 47) and controls (n = 25), followed by FDR correction. All the SE regions with FDR p-value < 0.05 were considered as differentially methylated. Of the initial 3627 SEs, 159 were differentially methylated (DM) between 47 tumors and 25 normal samples in JHU HPV + HNSCC cohort. The SEs with higher methylation in tumors relative to normal (138 SEs) were referred to as hypermethylated, while the remaining (21 SEs) with higher methylation in normal were referred to as hypomethylated.

### Identification of in cis SE targets

Recent data suggest that SE effects can reach *in cis* targets up to one Mbp away through chromatin loop formation^3–5,8,10,14,42^. Therefore, all genes with transcription start sites within one Mbp from the SE region were considered potential targets of the SE^2,3^. For each differentially methylated SE (n = 159), a list of all potential targets was assembled. SE candidates that covered the same genomic coordinates due to the utilization of four different cell lines were treated individually.

### Promoter methylation detection

We defined the gene’s promoter region as the genomic interval 1500 bp upstream and 500 bp downstream of the transcription start site. The methylation of each promoter was assessed in the same way as the SEs (see *SE differential methylation detection*).

### Methylation to expression correlation analysis

#### Correlation between target gene expression and SE methylation

For each pair of a differentially methylated super-enhancer and a gene with TSS within 1 Mbp of the SE region, we tested the hypothesis that the SE methylation regulated the gene expression. This hypothesis was tested by calculation of correlation (negative concordance) between the RSEM-estimated expression of the gene in each sample and DNA methylation of the SE region in the same sample for all samples in the JHU cohort^32,33^. We applied Kendall’s tau test using the Kendall package for R, version 2.2^53^. We used rank-based statistics to compare gene expression (measured by RNA-Seq) and DNA methylation (quantified by MBD-Seq or Illumina 450 k array). These data types produce different values and cannot be compared directly, only through their ranks. FDR-corrected Kendall’s tau test p-value < 0.05 was considered significant to link SE methylation and gene expression. Then, we filtered out all gene-SE pairs with positive correlation as artifacts. The remaining pairs were then considered separately for hypo- and hyper-SEs.

#### Correlation between target gene expression and promoter methylation

For all the target genes (with TSS in 1 Mbp from a DM-SE) of a SE, we also estimated the Kendall’s tau rank correlation between promoter methylation and gene expression to test whether the gene expression is regulated by promoter methylation. Similar to SEs, we used the Kendall package for R^53^ and FDR-corrected Kendall’s tau test p-values < 0.05 were considered significant.

### TCGA validation

We used 22 HPV + OPSCC and 6 normal samples from TCGA^21^ that have both Illumina 450 k DNA methylation and RNA-Seq expression data to validated gene-SE pairs. For every pair, we found Illumina probes in a SE region and applied Kendall’s tau test^53^ to methylation beta values of these probes and the gene expression values of potential target genes. For every SE-gene pair for which the SE region has at least one Illumina probe, we found the probe with the minimum correlation coefficient with the gene expression value. A pair was considered as validated if Kendall’s test p-value for the probe was less than 0.05.

### Overrepresentation gene set analysis

Overrepresentation gene set analysis was done by computing overlaps with annotated Hallmark gene sets in MSigDB v6.1^54^.

## Supplementary information

Supplementary Information

Supplementary Tables
