# Supplementary material for: Differentially Methylated Super-Enhancers Regulate Target Gene Expression in Human Cancer
Source: Sci Rep. 2019 Oct 21;9:15034. doi: 10.1038/s41598-019-51018-x (PMC6803762; doi:10.1038/s41598-019-51018-x)
Supplement: Supplementary file 1 — Supplementary Information [file 41598_2019_51018_MOESM1_ESM.pdf]

Title: Differentially Methylated Super-Enhancers Regulate Target Gene Expression in Human Cancer

Emily L. Flam, Ludmila Danilova, Dylan Z. Kelley, Elena Stavrovskaya, Theresa Guo, Michael Considine, Jiang Qian, Joseph A. Califano, Alexander Favorov, Elana J. Fertig, Daria A. Gaykalova

SUPPLEMENTARY FIGURES

Hypermethylated SE: chr12:52622299–52631702
TCGA cohort

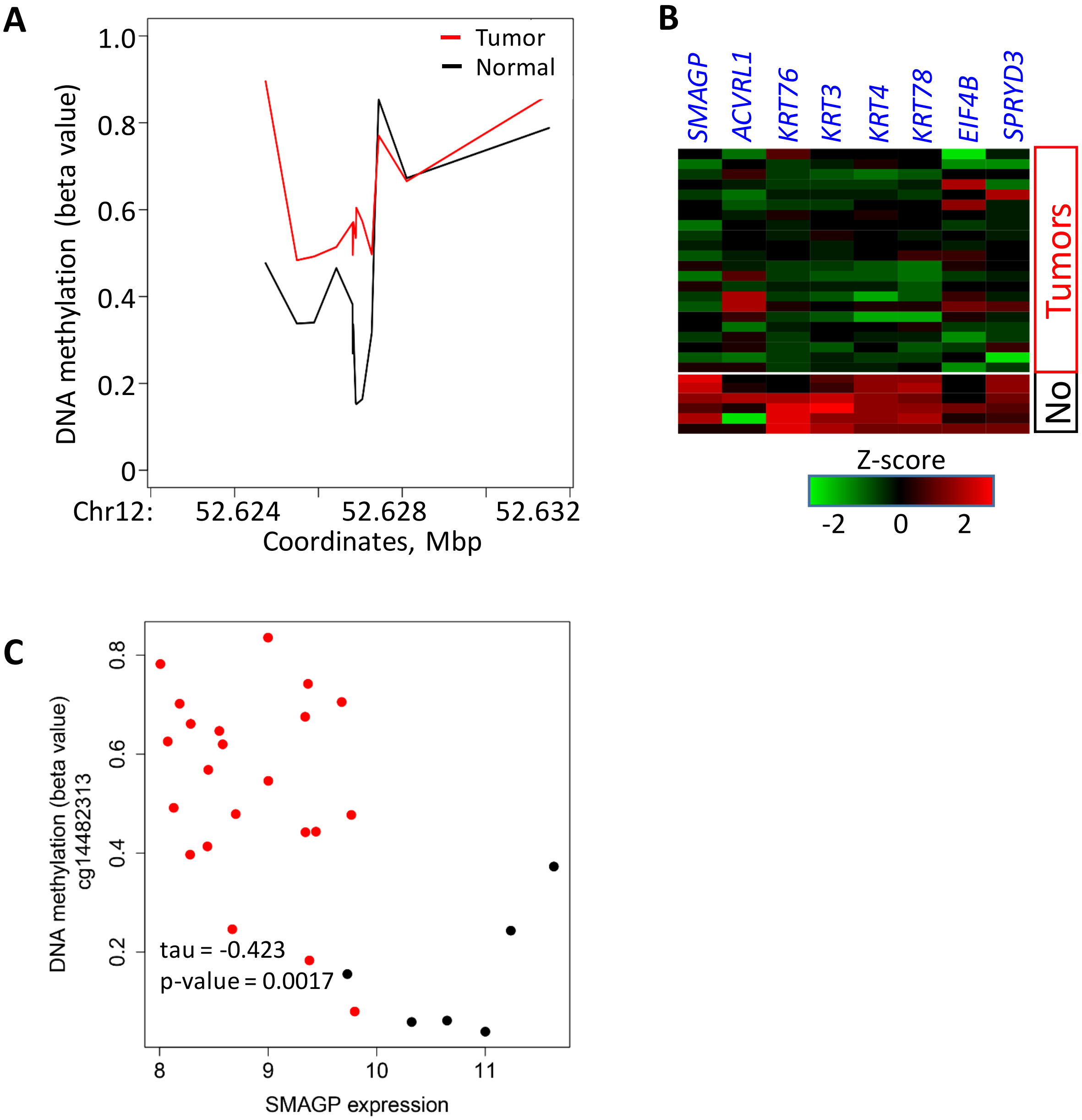

Figure S1. Methylation and genetic landscape of hypermethylated SE: chr12:52622299–52631702 in TCGA cohort. A) Relative average methylation coverage across SE region. B) Log-transformed RNA expression of genes within one Mbp of SE region (z-score). C) log-transformed gene expression of *SMAGP* vs. SE methylation.

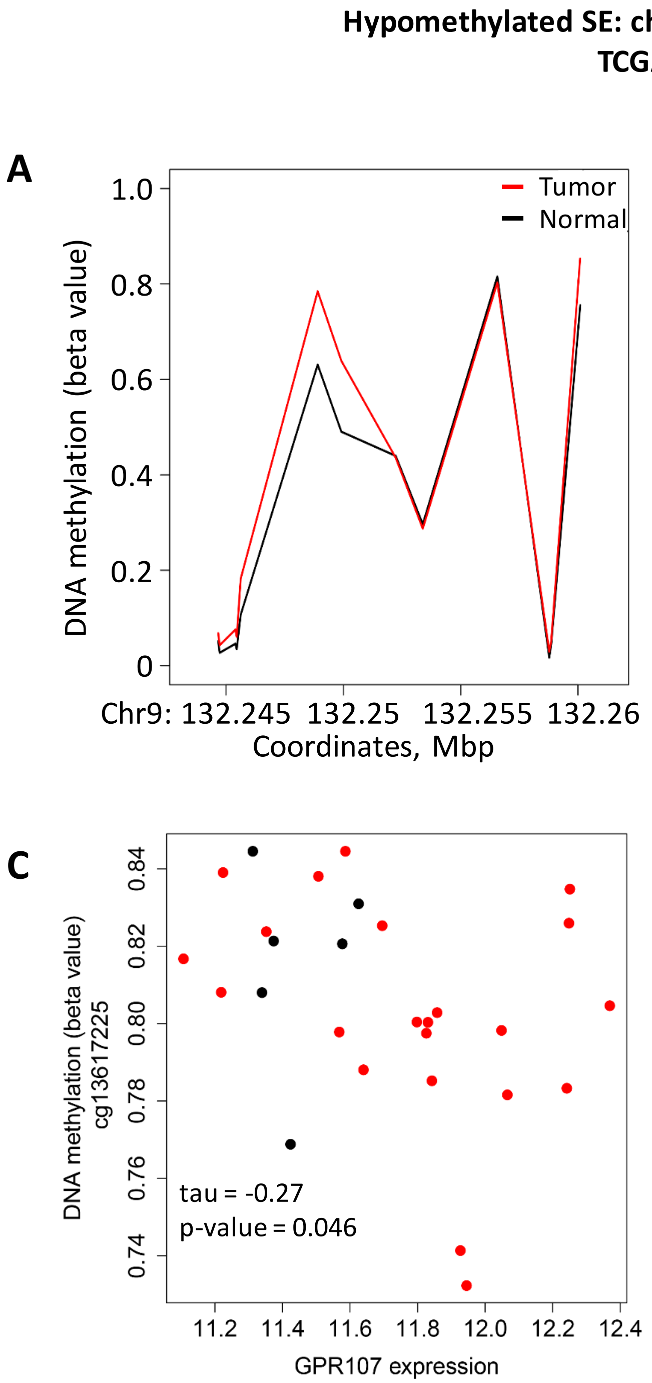

Figure S2. Methylation and genetic landscape of hypomethylated SE: chr9:132243320–132261430 in TCGA cohort. A) Relative average methylation coverage across SE region. B) Log-transformed RNA expression of genes within one Mbp of enhancer region (z-score). C) log-transformed gene expression of *GPR107* vs. SE methylation.

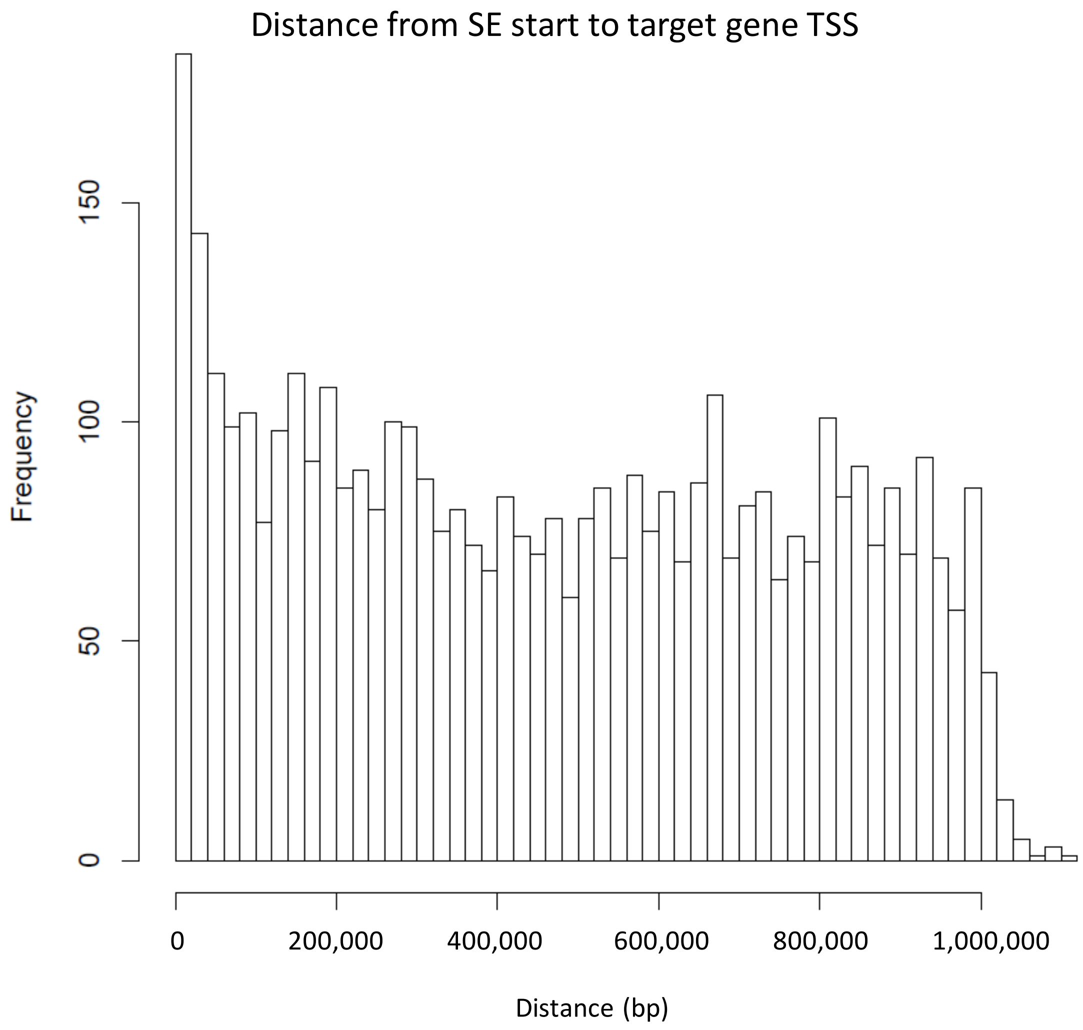

Figure S3. Distance from SE start to target gene TSS. The distribution plot of the distance of identified target genes from the start of DM-SEs.

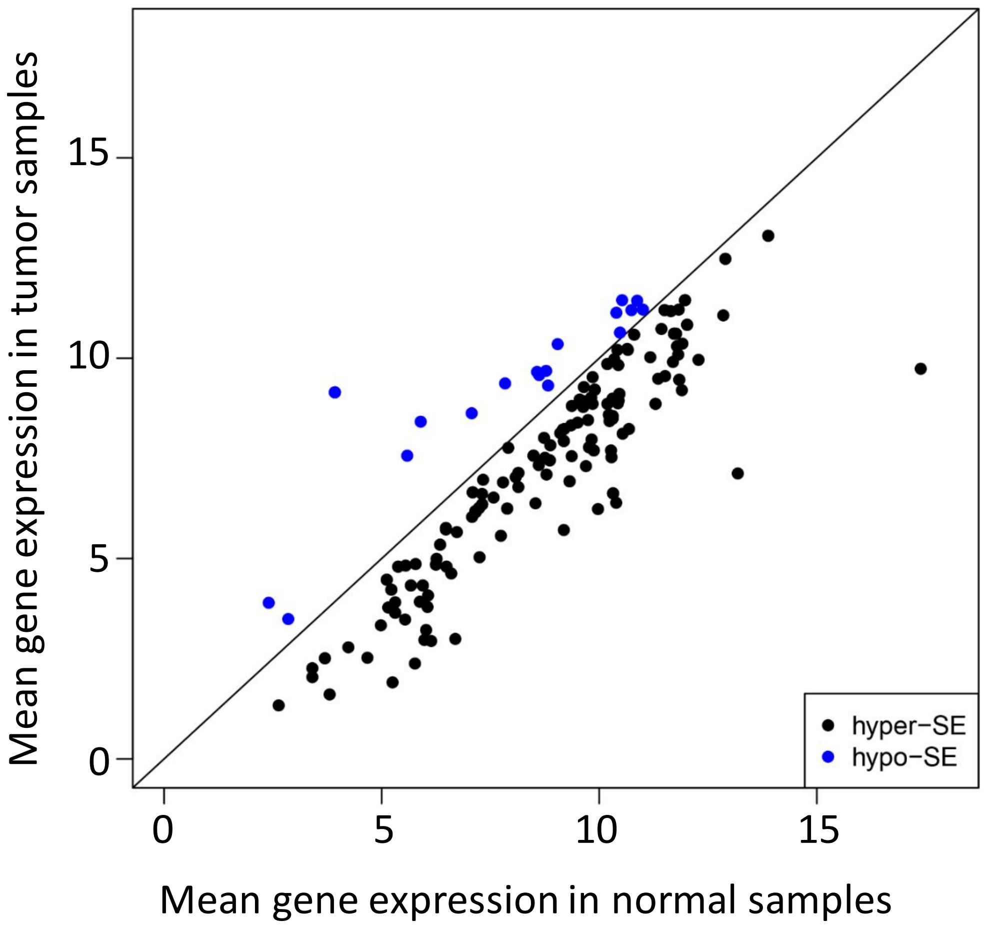

Figure S4. Average gene expression trends of SE target genes. Log-transformed gene expression in normal samples vs. tumor samples in differentially expressed target genes of hypermethylated SEs (black) and hypomethylated SEs (blue).

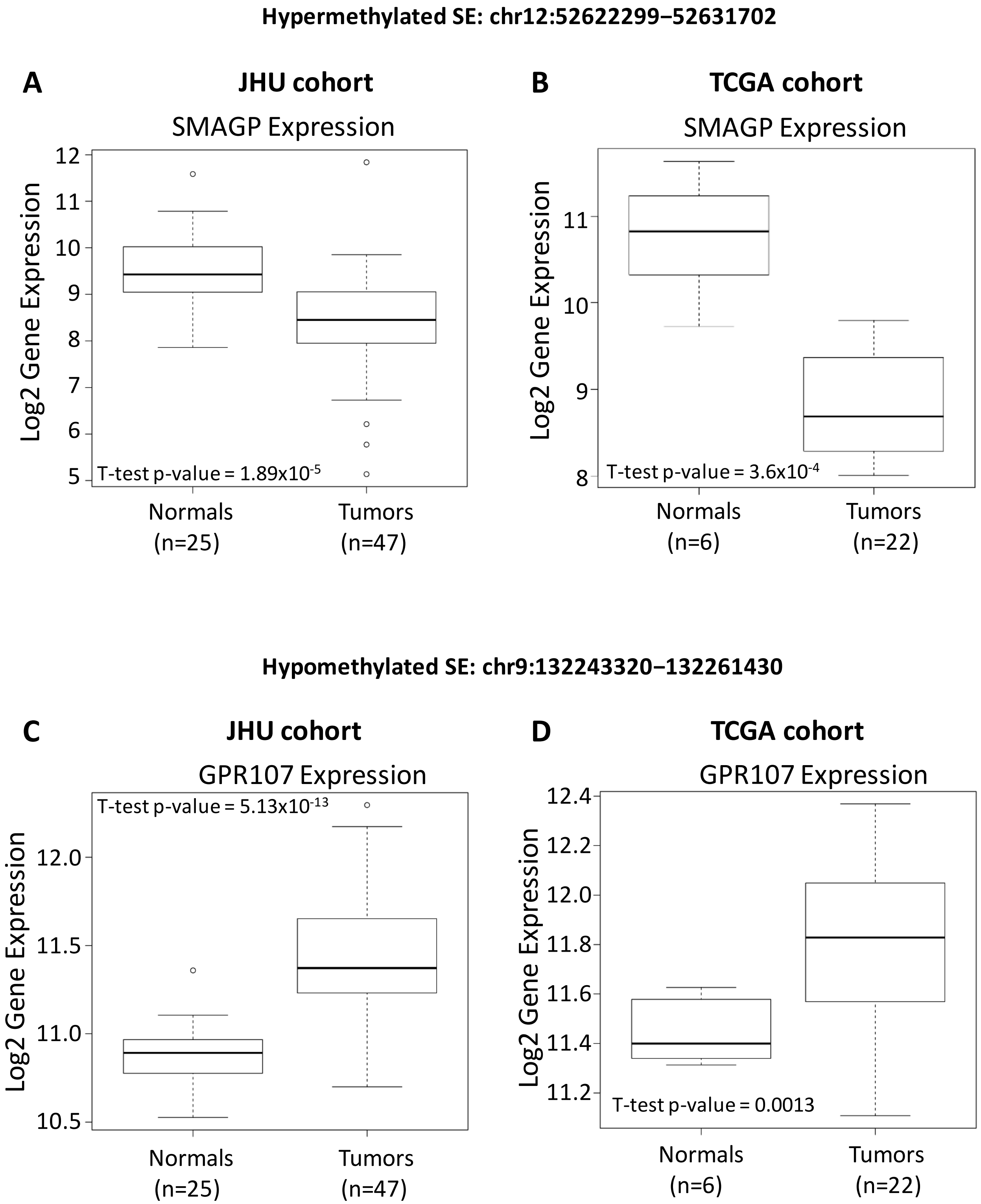

Figure S5. Gene expression of enhancer target genes. Boxplots of log-transformed gene expression in tumor vs. normal populations of Kendall-tau-validated target genes, *SMAGP* and *GPR107*, in JHU (A, C) and TCGA (B, D) cohorts.

SUPLEMENTARY TABLES

Table S1. Study Samples. The description of samples, which SE lists were used as an input for our pipeline.

Table S2. Description of input SE candidates. The list of all input SEs from five study samples (Table S1), their location, length, samples source, the number of target genes, as well as DNA methylation status evaluated by Wilcoxon test.

Table S3. The output list of SE-gene pairs. The correlation of gene expression and SE or promoter methylation status in JHU or TCGA cohort

Table S4. MSigDB gene set enrichment analysis of 132 genes linked to hypermethylated SEs and 18 genes linked to hypermethylated SEs.

MAIN PIPELINE DESCRIPTION

Differentially Methylated Super-Enhancers Regulate Target Gene Expression in Human Cancer

May 02, 2019

Table of Contents

INPUT FILES 8
SCRIPT: 8

INPUT FILES

5CellLines_SEs.csv contains 3627 SEs from five cell lines (H2171,UCSD_Lung, HeLa, NHLF, IMR90) from the Hnisz et al. paper (PMID: 24119843)

Download, unzip, and copy RNAseq data from GEO (GSE112026) to the root folder with scripts: https://www.ncbi.nlm.nih.gov/geo/download/?acc=GSE112026&format=file&file=GSE112026%5FHPVOP%5FRSEMNorm%2Etxt%2Egz

Install differential.coverage, the package to work with MBDseq data (https://github.com/favorov/differential.coverage):
devtools::install_github("favorov/differential.coverage")

The data folder contains all necessary files to get methylation data using the differential.coverage package. BED files are the results of peak callings from ROSE. The raw data can be downloaded from GEO (GSE112023): https://www.ncbi.nlm.nih.gov/geo/query/acc.cgi?acc=GSE112023

sampleID_mapping.txt contains mapping between IDs of samples with RNAseq and MBDseq data

SCRIPT:

The R code of the pipeline is available by this link: https://bitbucket.org/favorov/cervical-lung-se-and-hnscc/downloads/.

```
#==================
# parameters

# interval for promoter methylation
tssUp = 1500
tssDown = 500
```

```
# range of SE region +/- seFlank to find target genes
seFlank = 1e+6
# significance thresholds
dmFDR = 0.05
corFDR = 0.05

#==============================
# run scripts

# loads all necessary data
source('loadData.r')
# calculates differential enhancer methylation
source('diffMeth.r')
# calculates correlation of promoter/enhancer methylation and gene expression
by Kendall tau
source('kendallCor.r')

#=========================
tumSampExpr = rownames(sampAnnot)[which(sampAnnot[,'class']=='T')]
normSampExpr = rownames(sampAnnot)[which(sampAnnot[,'class']=='N')]
# get average expression in tumors and normal and direction of expression in
cancer
geSummary = cbind(normal_mean_expr = apply(rnaData[,normSampExpr],1,mean, na.
rm = T),
    tumor_mean_expr = apply(rnaData[,tumSampExpr],1,mean, na.rm = T))
geSummary = cbind(geSummary, Expr_in_cancer = apply(geSummary,1, function(x)
if(x[1]<x[2])return('up')else return('down')))

# combine all data and create BigGeneTable
BigGeneTable = cbind(enhMethCorPval, # correlation of enhancer meth and gene
expr
    dmTable[enhMethCorPval[,'enhancer'], ], # differential methylation of enh
ancers
    data.frame(promMethCorPval)[enhMethCorPval[,'gene'],], # correlation of p
romoter meth and gene expr
    geSummary[enhMethCorPval[,'gene'],]) # differential expression
#===================
# subset big table
# significantly differentially methylated SE
hyperBigTable = BigGeneTable[which(BigGeneTable[,'meth.status'] == 'hyper'& a
s.numeric(as.character(BigGeneTable[,'wilcox.FDR']))< dmFDR),]
hypoBigTable = BigGeneTable[which(BigGeneTable[,'meth.status'] == 'hypo'& as.
numeric(as.character(BigGeneTable[,'wilcox.FDR']))< dmFDR),]

# find pairs that have FDR < 0.05 (significant Kendall tau) and negative corr
elation
hyperBigTable_05 = hyperBigTable[which(as.numeric(as.character(hyperBigTable[
, 'EnhancerFDR'])) < corFDR &
    as.numeric(as.character(hyperBigTable$EnhancerCor)) < 0),]
hypoBigTable_05 = hypoBigTable[which(as.numeric(as.character(hypoBigTable[, '
```

```r
EnhancerFDR'])) < corFDR &
    as.numeric(as.character(hypoBigTable$EnhancerCor)) < 0),]

# final set of SE-pairs to validate
write.table(rbind(hyperBigTable_05,hypoBigTable_05), file='geneEnhancerPairs_
toValidate.txt', sep="\t", row.names = F)

print(sessionInfo())
```

```
## R version 3.5.0 (2018-04-23)
## Platform: x86_64-w64-mingw32/x64 (64-bit)
## Running under: Windows 10 x64 (build 17134)
##
## Matrix products: default
##
## locale:
## [1] LC_COLLATE=English_United States.1252
## [2] LC_CTYPE=English_United States.1252
## [3] LC_MONETARY=English_United States.1252
## [4] LC_NUMERIC=C
## [5] LC_TIME=English_United States.1252
##
## attached base packages:
## [1] stats     graphics  grDevices utils     datasets  methods   base
##
## loaded via a namespace (and not attached):
##  [1] compiler_3.5.0  magrittr_1.5    tools_3.5.0     htmltools_0.3.6
##  [5] yaml_2.2.0      Rcpp_1.0.1      stringi_1.4.3   rmarkdown_1.12
##  [9] knitr_1.22      stringr_1.4.0   xfun_0.6        digest_0.6.18
## [13] evaluate_0.13
```
